# Recognising values and engaging communities across cultures: towards developing a cultural protocol for researchers

**DOI:** 10.1186/s12910-021-00608-4

**Published:** 2021-04-26

**Authors:** Rakhshi Memon, Muqaddas Asif, Ameer B. Khoso, Sehrish Tofique, Tayyaba Kiran, Nasim Chaudhry, Nusrat Husain, Sarah J. L. Edwards, Pakistan Institute of Living and Learning (PILL), Nasim Chaudhry, Afshan Qureshi, Shela Minhas, Tayyeba Kiran, Ambreen Khan, Sehrish Tofique, Maryam Tahir, Munazzah Farooq, Ashfaq Ahmed, Rab Dino, Maria Usman, Nawaz Khan, Tahira Khalid, Sehrish Irshad, Rabia Sattar, Akhtar Zaman, M. Asif, Ayesha, Imrana Imrana, Sanaullah, Shafaq Ejaz, Umair Ahsan, Zainab Bibi, Amna Noureen, Anum Naz, Uzma Omer, Farhatulain, Awais Khan, Suleman Shakoor, Muqaddas Asif, Maham Rasheed, Usman Arshad

**Affiliations:** 1 University College London, London, UK; 2 Pakistan Institute of Living and Learning (PILL), Karachi, Pakistan; 3 University of Manchester, Manchester, UK; 4 Honorary Consultant Psychiatrist Lancashire & South Cumbria NHS Foundation Trust, Preston, UK

**Keywords:** Bioethics, Community engagement, Global health, Low and middle income countries (LMIC), Cultural protocol, Researchers from high income countries (HIC)

## Abstract

Efforts to build research capacity and capability in low and middle income countries (LMIC) has progressed over the last three decades, yet it confronts many challenges including issues with communicating or even negotiating across different cultures. Implementing global research requires a broader understanding of community engagement and participatory research approaches. There is a considerable amount of guidance available on community engagement in clinical trials, especially for studies for HIV/AIDS, even culturally specific codes for recruiting vulnerable populations such as the San or Maori people. However, the same cannot be said for implementing research in global health. In an effort to build on this work, the Pakistan Institute of Living and Learning and University College London in the UK sought to better understand differences in beliefs, values and norms of local communities in Pakistan. In particular, they have sought to help researchers from high income countries (HIC) understand how their values are perceived and understood by the local indigenous researchers in Pakistan. To achieve this end, a group discussion was organised with indigenous researchers at Pakistan Institute of Living and Learning. The discussion will ultimately help inform the development of a cultural protocol for researchers from HIC engaging with communities in LMIC. This discussion revealed five common themes; (1) religious principles and rules, (2) differing concepts of and moral emphasis on autonomy and privacy, (3) importance of respect and trust; (4) cultural differences (etiquette); (5) custom and tradition (gift giving and hospitality). Based on the above themes, we present a preliminary cultural analysis to raise awareness and to prepare researchers from HIC conducting cross cultural research in Pakistan. This is likely to be particularly relevant in collectivistic cultures where social interconnectedness, family and community is valued above individual autonomy and the self is not considered central to moral thinking. In certain cultures, HIC ideas of individual autonomy, the notion of informed consent may be regarded as a collective family decision. In addition, there may still be acceptance of traditional professional roles such as ‘doctor knows best’, while respect and privacy may have very different meanings.

## Background

The debate on the ethics of international clinical research involving collaboration with Low and Middle Income Countries (LMIC) is of considerable significance because of increased interest in global health research [1]. This makes it an imperative that research is equitable, just, relevant to the context in which it is organised and responsive to local health needs. Global health ethics is emerging as a new discipline [2, 3]. Although the field of global health ethics is largely underpinned by the concepts of bioethics from HIC, it is increasingly important to examine such concepts in different cultural and social contexts. Concerning ethics, Bernal and Adames [4] stated that “we caution to impose views, norms and values of the world’s dominant HIC society onto vulnerable populations such as ethno-cultural groups”. Considered in the round, the discipline of research ethics is mostly ‘Euro-American’ and not often recognised, let alone adapted for, within many other cultures [5, 6]. For example, Zaman and Nahar note that when conducting research in Bangladesh, they found the word ‘research’ did not exist in the Bengali language and when translated meant ‘finding a lost cow’. Global health projects have too often been developed without the input of LMIC local partners, leading to claims of neo-colonialism [7]. The conditional adherence to HIC governance models by the funders of research who are predominantly from the global north could similarly be seen as neocolonial.

In defining “Global Health Ethics” Myers [8] likewise observed “that existing literature on global health ethics has majorly originated from north American medical doctors. This is corroborated by random searching of literature using the terms ‘community engagement’, ‘global health ethics’, ‘global health research and ‘global health partnerships’ from 2016 to 2020 revealed around 102 articles mostly from HIC. This was inferred by the first author’s names. Therefore, the question of whose perspectives, views, plans as well as advances, the global health ethics actually represents and how and why—invites scrutiny” [8]. As Japanese historian William LeFleur once commented, though, “bioethics has become international, it has not become ‘internationalised” [9]. HIC principles of bioethics focus on the individual’s rights of autonomy and consent disregarding the collectivistic cultural norms of interconnectedness of role of the family and how shared decision making is intertwined in some cultures. Consequently, despite all good intentions towards research participants, researchers possess a certain ‘ethnocentrism’ before research begins. While research carried out in a respectful manner has maximised social value [10], community engagement or community consultation in the proposed research projects has emerged as a requirement for ethical international research [7]. It refers to participation and involvement of people, groups, structures or community members for planning, design, decision-making, and governance to promote people centred delivery of services [11]. It is recently seen as critical and fundamental component of many health initiatives, particularly during disease outbreaks [12]. This entails understanding of the emergent global health gaps and of the lifestyle of potential research participants. In their book, Understanding Global Health, Velji and Bryant [13] state that when conducting research, global health ethics not only challenge its practitioners to identify potential research subjects but to assure respect for justice, dignity and human rights. It is necessary to understand the different mind sets, environments and frameworks of thinking while undertaking collaborative research in LMIC [2]. Kolev and Sprowl [14] emphasised the importance of addressing those aspects of health systems that continue to hinder efforts to meaningfully engage with patients, their families and local communities. In today’s world, when researchers may not reside in the communities where they work, knowing their experiences and culture i.e. differences in political, cultural and social structures, systems and processes among communities, social norms and beliefs is important.

With the World Health Organisation (WHO) global health agenda [15] and the UN Sustainable Development Goals (SDG’s) of 2030 [16] more and more young researchers want to apply their skills to global health research. It is a good thing that more and more research is being carried out in the pursuit of global health and that researchers and institutions from HIC are invited to build research capacity and capability. As part of these projects, researchers from HIC are often involved in community engagement workshops. In culturally diverse environments where linguistic and cultural barriers exist, standards for effective communication might be daunting [2, 3]. Researchers from HIC are often unsure of the cultural norms, values and beliefs of local communities. Consequently, they may sometimes unknowingly come across as insensitive and disrespectful. **“**How do we begin to think about unintended consequences when we are doing what we presume as ‘good’ for the patient, for their family, the community and society at large” [12].

Implementation of health research requires understanding and engaging key stakeholders at all levels of the local health systems. Cultural and linguistic variances, historic legacy of mistrust, manipulation within the research enterprise and scientific colonisation concerns further intensifies these conditions [17]. MacLachlan [18] highlighted how communities may see foreign aid workers as symbols of colonialism, capitalism, and eurocentrism. Conversely, communities may perceive the doctor from HIC to have magical powers and superior expertise. This can give rise to unrealistic expectations. To counter this, honesty must be a universal commitment [12].

Existing literature in research ethics has grappled with differences in culture to the extent that codes of conduct are now being developed to redress the balance. For example, the San Code of Research Ethics is written with the San people of South Africa for all research involving them [19]. Another code, the Te Ara Tika—Guidelines for Maori Research Ethics: A Framework for Researchers and Ethics Committee Members seeks to secure sensitivity to culture in the structural protections of research participants [20]. Community engagement guidance in clinical trials is available, particularly for HIV/AIDS in Africa [21] but there is a gap in guidance for implementing research in global health. To make interventions more relevant and meaningful to local people, whichever culture they are from, community participation has been regarded as essential.

Building on existing work in clinical trials and for research with specific cultures, thought to be vulnerable and in need of special consideration, more clear and specific guidance is needed for incorporating effective and relevant community engagement methodologies into planning and implementation [22]. Such guidance can help implementation researchers to engage community stakeholders in more strategic and practical ways to enhance quality and meaningful application of results in order to improve health and health system outcomes. This would not only assist planning for specific projects but would also be a useful contributor to ensure that new and early career researchers are better equipped to consciously and thoughtfully engage with communities affected by their work in ways that are respectful and empowering [22].

How we should think about differences in values between cultural and wider communities is a familiar topic to philosophers, yet the limits to cultural and moral relativism in global health research is yet to be resolved. If communities and cultures across the world were to have very different values, it would be almost impossible to find common ground and global health research would be effectively impossible to pursue. Rather than to start with relative moral values, it is possible to converse with members of different cultures about their values and to devise a ‘bottom up’, or rather a participatory framework informed by experience of global collaborations in the field. As a contribution towards moving forward the global health research endeavours, development of a cultural protocol would be a fundamental starting point to help dispel false preconceptions and improve communication for a better understanding of the research environment and its people. The essence of a participatory approach is to recognise ‘that people whose lives are to be changed by developing interventions should have a say in what these changes are to be, and how they will take place’ [23]. Although, there are various ways and approaches designed to facilitate stakeholder engagement at the national or institutional level [22], these tools are intended to assist as aids to discussion around community involvement issues within a particular partnership and/or as a way of exploring comparisons between partnerships. These are at a very early stage of development and have not yet been tested on the ground [24].

Pakistan Institute of Living and Learning (PILL) has many visiting researchers from HIC, who are involved in community engagement, training and development. PILL and University College London (UCL), UK have collaborated to identify the gap and to gain an understanding of the beliefs, cultural norms, and values of local communities with the aim to better understand and manage the differences in values and ethics in a different cultural setting. The rationale underpinning the development of a cultural protocol is that when there is no clear communication and understanding of the beliefs, culture, norms, nuances and values of a particular community, there is no foundation on which to build rapport, respect and trust needed for initiating the process of community engagement itself. If we believe in local participation and community engagement to be core values underpinning participatory approach, we need to make a concerted effort to gain insight into the belief system, norms and values of the communities we work with.

A culture protocol for researchers is not intended as a substitute for community engagement which itself is an important process for eliciting views and values of particular communities and relates to particular research proposals and projects. A cultural protocol, however, would prepare researchers to approach the process of community engagement with respectful cultural sensitivity. The aim of this paper is to present findings from a discussion group towards development of a cultural protocol for researchers for community engagement in Pakistan.

## Methodology

This paper is based on a discussion group with local indigenous researchers from Pakistan.

### Setting

The discussion group was convened on January 11th, 2019 at Pakistan Institute of Living and Learning (PILL) head office in Karachi with four other PILL centres from different cities via zoom. The discussion was in English and Urdu to facilitate communication. The participants were researchers from PILL (n = 32), they were all qualified psychologists who had experience of community engagement and partnership relied research activities across Pakistan and had volunteered for the group discussion. The discussion group lasted for one hour and 28 min. Written consent was taken from all the participants to audio record the discussion and to use the materials for publication.

A semi structured list of questions to facilitate the discussion was developed following literature review. This explored researcher cultural beliefs and the impact on their research activities, challenges and responsibilities as a researcher coming from another country. The discussion was moderated by RM (based in UK with a background in health services management and improvement sciences). The group discussion revolved around the experiences and learning of local researchers from community engagement activities.

### Analysis

The audio recording of discussion was transcribed by a qualitative researcher. The transcription was translated into English by a bilingual researcher. Translated transcript was then back translated into Urdu for checking by an independent researcher. Thematic analysis was used to analyse the discussion [25]. Steps involved in the analysis included familiarisation, generating initial codes, searching for themes, reviewing themes, defining and naming themes and finally transforming it in a report form. RM and MA did initial line by line coding and finalising the themes both were supervised by an experienced qualitative researcher (TK) to maintain data credibility and trust worthiness. TK reviewed the transcript and analysis.

## Results

The results presented here are based on the general themes that emerged from the discussion. The five themes from the discussion include: (1) religious principles and rules, (2) concept of autonomy and privacy, (3) notion of respect and trust, (4) cultural differences (etiquette) and (5) custom and tradition (gift giving and hospitality). The five themes which emerged from the discussion are as follows and the key messages from these five themes are in Table 1.

1. Religious principles and rules

**Table 1:** Themes and key messages

No	Themes	Key messages
1	Religious principles and rules	Religious practices are a way of life in the Pakistani culture Prayer times should be respected, particularly Jummah (Friday) prayers. Men in particular go to the mosque to pray Imams and other religious scholars are held in high esteem in their communities. Building rapport with these religious leaders would help
2	Concept of autonomy and privacy	The notion of ‘shared decision making’ translates into the whole family being involved in deciding on what care should be given to the patient
3	Notion of respect and trust	Segregation between genders is expected and respect for female family members, stems from the religious and traditional belief to protect the family Strong family loyalties and ties are based on the religious belief that looking after one’s own comes over and above any other relationships
4	Cultural differences (etiquette)	Making eye contact and shaking hands across genders and age have different meaning
5	Custom and tradition (gift giving and hospitality)	Love and respect are expressed by giving gifts and offering food and drink to visitors Researchers from HIC may find this overwhelming. Saying no gently and politely would ensure people’s feelings are not hurt

Following the British colonial rule in India, Pakistan was founded in 1947 on the basis of religion; Islam. Hence, religion and culture are very much intertwined. Most societal norms are underpinned by religious beliefs. The community religious leaders once engaged can be a great ally. This was stated in the Group *“I think it is better to get hold of the imam in the community first. He prepares the ground for us by telling them who we are? And what we are trying to do? It makes our work much easier”.*

As Islamic traditions and practices are virtually ingrained in all parts of Pakistani life, it is an imperative to understand the significance of prayer, religious festivals and festivities have in everyday life. This was expressed several times in the discussion Group *“If it is prayer (namaaz) time specially Friday (Jummah) prayer we should not work during that time. Also, late afternoon (Asar prayer) and at sunset (Maghrib prayer). These prayer times are important so there should not be a clash with these times”.* Prohibition of food in Ramadan and music and other festivities during the month of Moharram were also mentioned *“During Ramadan people will be offended if you eat in public as everyone should be observing fast” “In Moharram, especially in Shia Sect we don’t play music or watch movies as it is the month of mourning”.*

Islamic ideals and customs were further reiterated. A discussant mentioned “*We do Qurbani (slaughter of an animal in the name of Allah) on Eid ul Adha. It may be shocking for foreigners but it is our religion, we do it in the name of Allah”.*

(2) Concept of autonomy and privacy

It would be quite usual for the whole family to be involved in discussions and decision making on issues which in the HIC maybe perceived as violation of autonomy. The tension between the primacy of autonomy in HIC bioethics literature is sometimes in contrast and incompatible with the social value system of interconnectedness and interdependence within cultures such as Pakistan which are religiously grounded. *“The whole families come and attend clinic with the patient. When people travel from far away villages, they have to camp outside hospitals for days as it is too costly and too far to go home every day”.*

Respect for the elders of the family and especially the husband in a male dominated society make ‘surrogate’ decision making an expectation and the norm. *“….. People come with their mother, husband, sometimes also friends. Mostly husband or mother will speak for them and decide.”*

(3) Notion of respect and trust

Distrust still exists within the (illegal) migrant community fleeing from Afghanistan to Pakistan since the Afghanistan war, as they may not possess identity cards. Young men dressed in foreign clothes are perceived to be government officials. “*So again it is related to credibility, integrity and trustworthiness. We experience in Orangi Town (in Karachi) that they don’t trust us. Actually as we are young men dressed in foreign clothes so they think that we are someone else, I mean they think we are ISI (Intelligence Services) or like that”.*

As segregation still exists in most families, traditionally male researchers will be restricted from home visiting. This was expressed by a discussant *“Only females can visit homes, male have to wait outside”.*

However, being respectful to customs and traditions goes a long way to build trust. As someone in the Group said *“if you sit on the chair and others are sitting on the floor, it is not good. It is all about respect”.*

Family and family loyalties are held sacred in the Pakistani culture and social fabric of the society. This value system is derived from the Islamic belief of individual and collective responsibility for the welfare of kin and kinship. This translates into family loyalty as long as the family member fulfils the criteria, be it nuclear or extended, family comes above other social relationships and even commercial arrangements. As one discussant in the group eluded “*My uncle who is a cloth merchant wanted me to manage his shop when I completed my university education".*

(4) Cultural differences (etiquette)

Eye contact and physical touching between genders is considered disrespectful and an invasion into privacy. Eye contact with the elders is considered to be challenging to the status of the elders and therefore also disrespectful and rebellious. The cultural polarisation of understanding of the same gestures between cultures can be perceived as disrespect, misunderstanding and offensive; this can be detrimental to community engagement and distrust of researchers from HIC.* “Making eye contact with your elders and opposite gender is considered disrespectful”.*

The segregation of genders historically, religiously and traditionally has required ‘Parda’ separation between male and female outside the family confines. This cultural conditioning and tradition continues. As observed by one discussant: *“Foreign professionals need to consider the cultural values before visiting other cultures. As most people don’t like to sit close to them or do hugs. Female professionals hesitate in sitting close with male foreigners”.* The point was further reinforced by another *“The girls don’t even shake hands with the boys, when they greet each other girls’ shake hands with each other but not with the boys”.*

A similar comment was made in the Group: *“You should prefer to wear long shirts with shawl and avoid wearing shorts or wear shawl or scarf. Is this relevant to female or is it relevant to male? Yes, both—So, they just should not go in shorts and t-shirts. Not shorts just jeans, trousers and shirts are best for male”.*

The dress code signifies not only adherence to cultural norms of modesty but also signals religious significance. *“I believe if people coming in foreign dress, they are seen as having more professional attitude, and coming with more professional background. They are having some kind of knowledge and people take them very seriously. Their comments and suggestions on any issue are taken very seriously”.*

Interestingly, personal space and boundaries are not the same as in the west. People may stand quite close when communicating. Also, albeit out of respect they may call male ‘bhai’ (elder brother) and female ‘baji’ (elder sister). As a result, they perceive that they now have a close enough relationship *“it is very common for people to become very friendly and ask very personal questions”* and *“they ask personal and intimate questions and also ask for personal numbers”.*

(5) Custom and tradition (gift giving and hospitality)

Pakistanis are renowned for their generosity and their love and respect towards their guests. In Islam, a guest is a blessing from Allah. So, however poor people are, they will go out of their way to welcome their guests with open arms. They believe that this would please Allah. *“I don’t know what foreigners do when they go for community field work but in our culture, they offer us tea, coffee, eggs, even seasonal fruits and many other gifts”.* Also, *“we have to take tea with them and have lunch when they offer us meal, to give them assurance that we belong with them and to win their trust”.*

## Discussion

Development of a cultural protocol for researchers would be a desirable step in fostering relationships between researchers from HIC and the communities they wish to recruit in research. In addition to accepted practices of community engagement and for special regard for protecting vulnerable cultures, observing a cultural protocol is another way in which researchers can show respect towards different cultural norms and values. A protocol would then become an integral part of community and participatory engagement process. We identified a number of key themes from discussion group including role of religious principles and rules, issues related to autonomy and privacy, notion of respect and trust, cultural differences in terms of etiquette and customs and traditions.

Previous evidence shows that involvement of religious leaders in raising awareness and community engagement is hugely important and they can play a significant role in bringing community on-board even when addressing taboo topic [26]. Therefore, engaging with and empowering religious leaders through capacity building and offering them support, recognition and appreciation can be an important component of community engagement [26]. Similar findings have been highlighted through this discussion group where researchers strongly emphasised the role of respecting religious practices and involving religious leaders to strengthen community involvement and trust.

An example of such a difference lies in the apparent need in HIC guidelines to protect the anonymity and confidentiality of participants [27]. However, this situation is different in Asian countries like Pakistan. In group discussion researchers emphasised the importance of shared decision making in Pakistani families. Accepting decisions made by elder members of the family is considered as matter of respect in Pakistani families and young people think that influence of elders is propitious for their life [28]. The joint decision making described in the results resonates with Dr Rose’s [12] narration in his book; A Blind Eye. He states ‘The Center is a buzz of clinical activity, like every facility in which I have worked in the developing world, the concept of privacy is wholly irrelevant. Observed by extended family, elders, and sometimes even village chiefs, several examinations take place in each room’.

Hospitality and gift giving is also considered as a universal behaviour in Pakistani culture that helps to integrate a society [29]. However, in a research context, gift giving poses an ethical challenge both for researchers and participants, since gift giving could be perceived by researchers from HIC as inducements, or worse, as bribes. In the discussion group, researchers highlighted that people in different communities express their hospitality by giving gifts. Therefore, people coming from other cultures should be aware of this gesture before visiting any community setting and be prepared to refuse gifts politely so that researcher-participant relationship is maintained.

In community based research, successful partnership between researchers and participants helps to generate trust and synergy [30]. Synergy in research is generated when participants hold space for a third culture that aims to integrate perspective of community members and academics, in order to generate innovative and valuable research [31]. Regarding trust Alpers [32] observed that ‘Cultural and linguistic differences may make it particularly challenging to build a trustful and positive relationship with patients of ethnic minority’. “Nepotism is seen positively as it assures hiring people who can be trusted” [33]. This would be an antithetical value stance from a HIC perspective. Attum, Waheed and Shamoon [34] in discussing cultural competence in the care of Muslim patients and their families state that “It is common understanding that women dress modestly. Men are mostly dressed to the knees or past the knees too. Although there is an impression that women dress modestly compared to men, but many men observe similar rules of modesty”.

Community engagement is concerned with directly approaching the host community and tapping into the community knowledge to identify the needs, issues and concerns. The community as the key stakeholder within the research process provides insight into the cultural and social context which is, as such, irreplaceable and invaluable to achieve the goals of the research. The enabling function of the protocol will be to inform and prepare researchers from HIC before they approach the community engagement process.

However, it is equally important to acknowledge that there are further sub-cultures within the different provinces of Pakistan so the development of a cultural protocol and guidance for community engagement with specific cultures will not only benefit visiting researchers from HIC it will also be of value to local indigenous researchers visiting different provinces within Pakistan.

## Conclusion

In summary, from available literature and discussion group, it has been clear that understanding local cultural values and norms are important to understand before initiating community engagement in health research primarily to facilitate recruitment. Underpinned by this reflective inquiry, development of a cultural protocol for researchers from HIC can be an important part of an overall engagement strategy in Pakistan. This could potentially provide pathways to develop a cultural protocol for researchers in LMIC to ensure local cultural norms and values for research are considered.

## Data Availability

A record of the full discussion used for the article is available from the corresponding author on reasonable request.

## Abbreviations

LMIC: Low and middle income countries
HIC: High income countries
PILL: Pakistan Institute of Living and Learning
UCL: University College London
